# Life situation of a caregiver of a child with congenital heart defect and/or other cardiac problems: structure and preliminary validation of a new questionnaire

**DOI:** 10.3389/fpsyg.2023.1194031

**Published:** 2023-06-15

**Authors:** Ewelina Kolarczyk, Aleksandra Morka, Kamil Barański, Lesław Szydłowski

**Affiliations:** ^1^Department of Gerontology and Geriatric Nursing, Faculty of Health Sciences in Katowice, Medical University of Silesia, Katowice, Poland; ^2^Department of Pediatric Cardiac Surgery, University Children’s Hospital, Faculty of Health Sciences, Jagiellonian University Medical College, Kraków, Poland; ^3^Department of Epidemiology, Faculty of Medical Sciences in Katowice, Medical University of Silesia, Katowice, Poland; ^4^Department of Pediatric Cardiology, Faculty of Medical Sciences, Medical University of Silesia, Katowice, Poland

**Keywords:** parents, caregivers, questionnaire, personal life, spirituality, children, congenital heart disease

## Abstract

**Background:**

Illness in a child with cardiac disease causes stress, brings additional responsibilities, reorganizes family life, and changes the functioning of the family.

**Aim:**

This study aimed to validate a new questionnaire evaluating the life situations of caregivers/parents of children with congenital heart disease (CHD) and/or other cardiac diseases (OCD).

**Materials and methods:**

The questionnaire comprised 10 questions aimed at assessing the life situation of an ill child’s caregiver in two main areas: personal and spiritual. The total score of the questionnaire assessing the life situation of the caregiver of a child with a CHD and/or OCD can range from 0 to 32 points, with scores <26 indicating a poor, 25 to 32 indicating an average, and >32 indicating a good level of life situation in the personal sphere of the caregiver. The questionnaire was assessed using Cronbach’s alpha tests, and repeatability was assessed using Cohen’s Kappa test (retest) within a time interval of two to 4  weeks from the first measurement.

**Results:**

The research covered 50 respondents. Cohesion in the personal sphere obtained a satisfactory value of Cronbach’s *α* = 0.72, in the spiritual sphere: Cronbach’s *α* = 0.83, and the result common for both sections was: Cronbach’s *α* = 0.66.

**Conclusion:**

The Life Situation Assessment Questionnaire for caregivers of children with CHD and OCD is a reliable and homogeneous tool for measuring the functioning of parents in the event of a child’s illness.

## Introduction

Congenital heart disease affects approximately 1.35 million children (van der Linde et al., 2011), which is nearly 1% of all children in the world (Sjostrom-Strand and Terp, 2019). In Poland, the incidence of heart defects in children is approximately 3,000 annually. Globally, around 9 of 1,000 babies are born with heart defects, and one-third of them require surgery in the first days of life (Science in Poland, 2022). Children with congenital malformations are hospitalized more often; they require costly diagnostics, other treatment procedures, and rehabilitation, often lifelong (American Heart Association, 2014). Children with congenital heart disease (CHD) require constant medical, nursing, and therapeutic care for survival and proper development. The care of a child with CHD, apart from the therapeutic team, is provided by the child’s parents. American studies conducted among parents of children with special needs have shown the occurrence of physical fatigue, frustration, malaise, anxiety, and a lack of energy to perform household duties and social activities (Caicedo, 2014). Lawoko and Soares (2002) revealed that parents of children with CHD reported higher levels of psychological symptoms such as stress, anxiety, and depression, than parents of children without CHD. A diagnosis of a chronic illness in a child results in the reorganization of the entire family’s life (Perrin et al., 2012); it is often a source of shock and stress for all family members (Guz et al., 2020). Stress can impair the communication and behavior of parents, affecting the relationship and interaction of the parent with the child and other family members (Lisanti et al., 2017).

In its assumption, professional medical care covers not only the patient but also family members caring for an ill child. Therefore, determining the biopsychosocial state of parents—the informal caregivers of the patient—is fundamental in the holistic care of a child with CHD. Extensive research has been conducted on the burnout (Hendrix et al., 2016; Riffin et al., 2019) and quality of life (Badia et al., 2021; Martin et al., 2021) of caregivers of people with chronic diseases. However, there is a lack of research in this area in relation to caregivers of children with CHD. Tools that provide information on emotions, family life, spiritual life, lifestyle, and the type of support needed by caregivers without medical education, and taking care of a child with CHD are lacking. When life’s difficulties increase, the need for spiritual support in spiritual terms becomes stronger, in the hope of finding solace in faith and beliefs (Ellis and Thomlinson, 2013). The home caregiver of an ill child is the first observer of ongoing therapeutic changes and provides emotional support and a sense of security to the child, which is conducive to the therapeutic process. Therefore, caregivers of children with cardiac problems should not be omitted from professional cardiac care.

Previous studies have shown that parents of children with CHD as their main caregivers require an individualized and multidisciplinary approach toward care to best adapt to the dynamic stressors associated with raising a child (Gramszlo et al., 2020). Emotional challenges related to being a caregiver of a child with CHD and/or other cardiac diseases (OCD) affect caregivers’ competence, well-being, and attachment to sick children (Knight Lozano et al., 2021). The literature on the subject has focused mainly on qualitative research on the functioning of parents/guardians of children with CHD and other OCD (Hoffman et al., 2020; Landry et al., 2022). However, there is a visible gap in research using standardized survey tools aimed at evaluating the functioning of parents/caregivers of children with CHD and/or OCD in terms of functioning in personal life and the role of spirituality in coping with life difficulties related to the child’s illness. In other studies (Coppola et al., 2021) it has been shown that those people who have declared to have a religious belief perceive greater levels of meaning of life and quality of relationships than those who have declared not to have religious belief.

The aim of the study was validated a questionnaire evaluating the life situation of caregivers/parents of children with CHD (and/or OCD). The study was conducted to assess the impact of challenges faced by caregivers/parents of an ill child. It focused on the functioning of the family, competence of caregivers, their well-being and attachment to the ill child in relation to the presented emotions, the assessment of the support received, and the importance of faith in God that allowed them to cope with a difficult life situation related to the child’s illness.

## Materials and methods

### Study design

This research was conducted at the Department of Pediatric Cardiology of the John Paul II Upper Silesian Children’s Health Center, a teaching hospital of the Medical University of Silesia in Katowice, Poland, from August 2022 to January 2023. The questionnaire was coded and distributed in paper form in two identical copies: The first copy was completed after submitting a written consent to participate in the study. The second copy was placed in an addressed envelope with a postage stamp, and given to the respondent to complete the questionnaire again after 2 weeks and send it back. The respondents also had the opportunity to complete the questionnaire again in an online version on Google Forms. The questionnaire was refilled within a strict period of two to 4 weeks from the first measurement, and was supervised by the researcher who stayed in touch with the respondents and reminded them via phone about completing and returning the questionnaire. The research was preceded by a pilot study conducted in July 2022 with 10 respondents. In accordance with the results of the pilot study, the instructions for the respondents on completing the questionnaire were clarified, and the language style of the questionnaire was modified for caregivers of both sexes.

### Participants

A total of 104 respondents participated in the study. However, since the second copy of the questionnaire was completed and sent back, only 50 respondents were finally included in the evaluation of the authors’ questionnaire. Responses were obtained from 90% (*n* = 45) of women and 10% (*n* = 5) of men. The average age of the respondents was 34.5 ± 5.1 years. Among women, the mean age was 34.3 ± 5.2 years, while that among men was 36.8 ± 3.3 years, the difference was statistically insignificant (*p* = 0.2). Detailed information about the study groups is presented in Table 1.

**Table 1:** Characteristics of the study group.

Parameter		*N*	%
Age [*years*]	M = 34.5 SD = 5.1		
Gender	Female	45	90%
	Male	5	10%
Education	Lack		
	Primary		
	Middle school	1	2%
	Vocational	4	12%
	Secondary	13	26%
	Higher	30	60%
Marital status	Single	1	6%
	Married	40	80%
	Widow/widower		
	Divorced		
	In a partnership	7	14%
Place of residence	Village	10	20%
	City ≤24,999 residents	6	12%
	City 25,000–49,999 residents	10	20%
	City 50,000–100,000 residents	8	16%
	City >100,000 residents	16	32%
Having other children apart from an ill child	Yes	32	64%
	No	18	36%
Professional activity	Employed on full time	25	50%
	Employed on part-time	3	6%
	Self business	4	8%
	Pension		
	Unemployment	18	36%
Unemployment	Lost the job		
	Quit the job because she/he wanted to	4	23.5%
	Quit the job because the situation forced her to do so	12	70.6%
	Takes a job for the first time		
	Returns to work after a long break	1	5.9%
Religion	Lack	6	12%
	Roman catholic	44	88%
	Other		
Religious practice	Believer and practicing regularly	17	34%
	Believer and non-practicing	12	24%
	Non-believer and non-practicing	6	12%
	Believer and practicing irregularly	14	28%
	Non-believer and practicing	1	2%

### Survey

The questionnaire used to assess the life situation of a caregiver/parent of a child with CHD (and/or OCD) was designed to facilitate the identification of factors determining the functioning of a caregiver/parent caring for a child with a cardiac disease, as well as to reflect the range of supporting factors in caring for an ill child. The self-report questionnaire comprises 10 questions aimed at assessing the life situation of a sick child’s guardian in two main areas: personal and spiritual. The questions refer to functioning in family, social, and professional life, and spiritual conditions that may indirectly affect functioning in difficult life situations related to a child’s cardiac disease. The responses were based on a five-point Likert scale: 5 points – “definitely not;” 4 points – “probably not;” 3 points – “neither yes nor no;” 2 points – “rather yes;” 1 point – “definitely yes.” For two questions—“Did your child’s illness make your family more united and family ties strengthened?” and “Did you stop believing in God because of your child’s illness?”—the responses were recorded as follows: 1 point – “definitely no;” 2 points – “probably no;” 3 points – “neither yes nor no;” 4 points – “rather yes;” 5 points – “definitely yes.” The total score of the questionnaire assessing the life situation of the caregiver of a child with a CHD and/or OCD can range from 0 to 32 points, where the scores <26 reflect a poor, 25 to 32 an average, and >32 a good level of life situation.

The questionnaire contained the following six descriptive questions: 1. Emotions of the caregiver/parent; the proposed answers were formulated on the basis of grouping the types of emotions into items proposed by Goleman (2005). A multiple-choice questionnaire survey was used. 2. Mental, spiritual, and material support for the caregivers/parents of a child with a CHD and/or OCD. The answer suggestions covered the areas of family, healthcare, religion, and other areas outside of those previously mentioned. This was a multiple-choice question and respondents could choose the person who supported them the most from among the listed suggestions. If the respondent could not indicate it, they could also choose “no one supports me” and “I do not need support.” 3. Causes of the child’s illness. It was an opinion-forming, multiple-choice question with the following proposed answer: “case;”“inheritance;”“punishment for sins;”“my neglect;“and “negligence of third parties.” 4. Identifying the person who plays the most important role in caring for the sick child. Detailed information regarding these questions is presented in Table 2.

**Table 2:** Multiple-choice descriptive questions of the survey questionnaire.

Question	Suggested answers *(multiple choice)*				
	A. Family area	B. Healthcare area	C. Spiritual area	D. Someone else like:	E. Other answer
Who supports you mentally (by lifting your spirits, showing empathy, caring) in dealing with your child’s illness?	Husband/wife partner my parents (mom and/or dad) my in-laws (father-in-law/mother-in-law) brother and/or sister	Physician nurse psychologist medical literature medical websites	Priest religious community/parish catechist nun support groups on religious websites	Friend colleague another person (e.g., uncle, aunt, etc.) online support groups	Nobody supports me I do not need support
Who supports you spiritually (help in finding the meaning of life, in suffering, in pain) in dealing with your child’s illness?					
Who supports you materially (financially) in dealing with your child’s illness?					
	If you have chosen more than one answer, please indicate which of the above-mentioned is the most important, if no one supports you (mentally/spiritually/materially) go to the next question				
How do you feel about your child’s illness?	Anger (resentment) sadness (regret, sad, despondent, discouraged) fear (anxiety, apprehension, concern) satisfaction (happiness) love (acceptance, trust, devotion) surprise (shock) disgust (contempt) shame (guilt, embarrassment, humiliation) none of the above hard to say other, what?.............................................				
Do you think your child’s illness is:	An accident inheritance of the disease “punishment for Sins” my neglect negligence of others hard to say Rather answer: ..........................................				
Who is the primary caregiver for an ill child?	The mother of the child the child’s father grandparents siblings of an ill child other person (e.g., uncle, aunt, etc..):……………………				

The last part of the questionnaire contained sociodemographic data, and the choice of answers was developed based on statistical data from the Central Statistical Office (2022) and the Public Opinion Research Center – Chancellery of the Prime Minister (CBOS communiqué, 2018). The sociodemographic questions and their answers are presented in Table 1. At the beginning of the survey questionnaire, the following information was provided to the respondent: “In this survey, we will address you in a direct personal form, the survey is completely anonymous and voluntary, and the results obtained will be used for scientific research, and this survey concerns the life situation of a caregiver/parent related to child’s illness.” The introduction also included instructions for choosing the most appropriate answer that the respondent thought of and which best reflected their opinion. If the respondent was not sure of the correct answer, the first answer they thought of should be given, as it was usually the closest to the truth. The questionnaire subject to this validation in its initial form also included five questions regarding the lifestyle of the caregivers/parents in connection with the child’s illness (Has their lifestyle changed? Has any physical activity been undertaken? Do they (caregivers) use drugs? Do they engage in risky behaviors? Do they eat properly?) and questions about the assessment of their well-being and economic and material situations.

### Statistical analyses

All statistical analyses were performed at the Statistics Department of the Medical University of Silesia, Katowice. The reliability of the measurement with the Life Situation Assessment questionnaire of the Caregivers of a Child with CHD and/or OCD was assessed with the use of Alpha Cronbach’s tests (internal consistency), and repeatability was assessed with the Kappa Cohen test (test–retest) over a period of 2 to 4 weeks from the first measurement. Differences in quantitative variables were assessed using the Wilcoxon test. All analyses were performed using the SAS statistical software (SAS Institute Inc., Cary, NC, United States, version 9.4.) The significance level was set at *p* < 0.05. In the assessment of repeatability, the following interpretation of Kappa Cohen statistics was used (Landis and Koch, 1977): <0.00 – no repeatability; 0.00–0.20 – poor repeatability; 0.21–0.40 – average repeatability; 0.41–0.60 – moderate repeatability; 0.61–0.80 – good repeatability; and 0.81–1.00 – very good repeatability. However, in the interpretation of the results of the Alpha Cronbach test, it was assumed that a value greater than 0.70 meant good internal consistency of the questionnaire (Cortina, 1993).

## Ethical approval

This study was approved by the Bioethical Committee of the Medical University of Silesia in Katowice (No.: PCN/CBN/0052/KB/44/22) and Hospital Management (L.dz./ZW/DO/DK/3787/22). Respondents were included in the study only after their written consent was provided; they were informed that the participation was voluntary and the lack of consent to participate would in no way affect the health services provided by the hospital to the child. In addition, the respondents were informed that if they consented to participate in this study, they would be able to withdraw at any time, without giving reasons, and failure to grant consent or its withdrawal would not entail any consequences for the respondent, in particular, the right to health care of their child.

## Results

To assess the repeatability of the questionnaire, key questions included in the questionnaire were considered. Table 3 lists the repeatability scores of the key questions in the questionnaire. In most cases, the agreement between answers exceeded 60% (from 60 to 80%). Repeatability analysis using Cohen’s Kappa statistics showed that the most repetitive questions concerned whether “the child’s illness made you give up on your own career plans and ambitions” – the Kappa Cohen value was 0.74, which means good repeatability, and this translated into 78% agreeing answers. A similar question was about faith in God – whether it was an important part of the respondent’s life. Cohen’s Kappa at the level of 0.71 proves good repeatability, which translated into 78% of consistent answers in the test–retest method. The weakest question in terms of repeatability of answers was about ceasing to believe in God because of the child’s illness; the value of the Kappa Cohen statistic was 0.25, which indicates average repeatability, but the agreement of the answers was 80%. The low value of Cohen’s Kappa is the result of the two respondents changing their minds that they did not stop believing in God in connection with the disease. A similar situation concerns the question of whether the respondent devotes less time to other children because of the sick child—Cohen’s Kappa 0.33, agreement 60%. Next, internal consistency was assessed in the context of the two scales in Tables 3, 4. The first assessed the impact of the child’s illness on various aspects of the respondent’s life. The most consistent question in this section was about the re-evaluation of family problems and the end of misunderstandings in the family as a result of the child’s illness *α* = 0.73, the least consistent question was about misunderstandings in the family as a result of the child’s illness *α* = 0.58. However, the overall consistency for the entire section yields a satisfactory value of 0.68. The detailed data are presented in Table 4. The next section discusses how religious beliefs changed because of the child’s illness. All questions achieved a satisfactory Cronbach’s *α* > 0.70, while the overall Cronbach’s *α* for the entire scale was 0.83. Detailed results are presented in Table 5.

**Table 3:** Assessment of repeatability of key questions included in the questionnaire.

Question	Kappa 95% PU	Repeatability
*Section of questions related to personal life*		
Has your child’s illness made you give up on your own plans and professional ambitions?	0.74 (0.60–0.88)	78%
Has your child’s illness made you limit your social life?	0.55 (0.34–0.75)	38%
Has your child’s illness made you limit your vacation trips, time for rest and pleasure?	0.58 (0.39–0.77)	70%
Has your child’s illness made your family more united and strengthened family ties?	0.42 (0.20–0.63)	62%
Has your child’s illness caused more misunderstandings in the family?	0.49 (0.27–0.70)	64%
Has your child’s illness made you pay less attention (time) to other children?	0.33 (0.09–0.57)	60%
*Belief in God/spirituality related questions section*		
Is believing in God an important part of your life right now?	0.71 (0.55–0.86)	78%
Are you convinced that faith in God and fervent prayer will restore health to your child?	0.52 (0.38–0.74)	64%
Have you stopped believing in God because of your child’s illness?	0.25 (−0.07–0.59)	80%
Do you pray more and participate in religious ceremonies because of your child’s illness?	0.53 (0.31–0.75)	72%

**Table 4:** Descriptive statistics and results for assessing the internal consistency of sections related to personal life.

Question	*n*	M ± SD	Me	Min–Max	Alpha Cronbach
Has your child’s illness made you give up on your own plans and professional ambitions?	50	2.70 ± 1.25	2	1–5	0.60
Has your child’s illness made you limit your social life?	50	2.68 ± 1.32	2	1–5	0.59
Has your child’s illness made you limit your vacation trips, time for rest and pleasure?	50	2.76 ± 1.33	2	1–5	0.59
Has your child’s illness made your family more united and strengthened family ties?	50	2.58 ± 1.18	2	1–5	0.64
Has the child’s illness caused more frequent misunderstandings in the family?	50	2.16 ± 1.15	2	1–5	0.58
Did your child’s illness make you reevaluate family problems and end misunderstandings in the family?	50	3.30 ± 1.05	3	1–5	0.73
Has your child’s illness made you pay less attention (time) to other children? (this question applies to people who have more than one child).	43	3.37	3	1–6	0.73

M, mean; SD, standard deviation; Me, median.

**Table 5:** Descriptive statistics and results for the assessment of the internal consistency of the spirituality section.

Question	*n*	M ± SD	Me	Min–Max	Alpha Cronbach
Is believing in God an important part of your life right now?	50	3.68 ± 1.00	2	1–5	0.72
Are you convinced that faith in God and fervent prayer will restore health to your child?	50	4.24 ± 0.98	2	1–5	0.75
Have you stopped believing in God because of your child’s illness?	50	4.90 ± 0.30	2	1–5	0.86
Do you pray more and participate in religious ceremonies because of your child’s illness?	50	3.16 ± 0.98	2	1–5	0.79

M, mean; SD, standard deviation; Me, median.

### Interpretation

**Figure 1:**
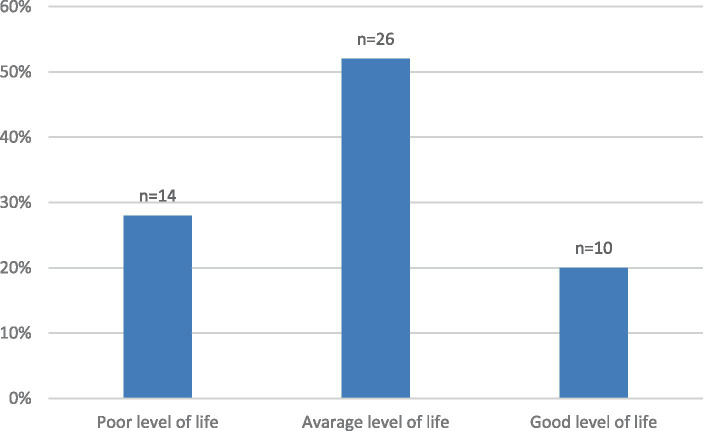
The score of the questionnaire assessing the life situation of the caregiver of a child with a CHD and/or OCD.

In the final stage of the study, a scale was used to measure the strength or level of the studied aspects. Taking into account the results related to the respondents’ answers, we assumed that a score below 26 points indicates a poor life situation, a score between 25 and 32 points indicates a good life situation, and a score above 32 points indicates a very good life situation. In the case of the first subscale evaluating the functioning in the personal life of a caregiver/parent of a child with congenital heart disease, scores below 11 points indicate a poor level, between 10 and 15 points a good level, and above 20 points are classified as a very good level. The second subscale describes the impact of faith in God/spirituality on the life functioning of a caregiver/parent in the event of a child’s illness; scores below 26 points indicate a poor level, between 25 and 32 correspond to a good level, and above 32 points indicate a very good level. The distribution of the total scores and results of the questionnaire subscales are presented in Table 6 and Figure 1.

**Table 6:** Distribution of answers.

	Frequency	Percent	Cumulative	Cumulative
			Frequency	Percent
SS1				
1	15	30.00	15	30.00
2	24	48.00	39	78.00
3	11	22.00	50	100.00
SS2				
1	15	30.00	15	30.00
2	27	54.00	42	84.00
3	8	16.00	50	100.00
SSA				
1	14	28.00	14	28.00
2	26	52.00	40	80.00
3	10	20.00	50	100.00

SS1, section of questions related to the personal life of a parent/caregivers of a sick child; SS2, section of questions related to faith in God/spirituality of a parent/caregivers of a sick child; SSA, two sections in total; 1,2,3, distribution of responses according to the adopted criteria of cut off points.

### Questions excluded from the questionnaire

Two questions related to personal life are excluded from this section. One of them was the question about well-being, which turned out to be too sensitive, with only 32% (*n* = 16) of agreeing answers. In this study, 38% (*n* = 19) of respondents rated their well-being as good, 34% (*n* = 17) as fairly good, 24% (*n* = 12) as neither good nor bad, and 4% (*n* = 2) as rather bad. The financial situation of 6% (*n* = 3) of the respondents was very satisfactory, 62% (*n* = 31) quite satisfactory, 22% (*n* = 11) neither satisfactory nor unsatisfactory, 6% (*n* = 3) rather unsatisfactory, and 4% (*n* = 2) said that it was unsatisfactory. The second question excluded from the section assessing the personal lives of parents of sick children was the one concerning the assessment of the economic and material situation. 6% (*n* = 3) of respondents answered that their situation was very satisfactory, 62% (*n* = 31) considered their situation rather satisfactory, 22% (*n* = 11) neither satisfactory nor unsatisfactory, 6% (*n* = 3) rather unsatisfactory, and 4% (*n* = 2) unsatisfactory. There was a 70% consensus response, and Cohen’s Kappa value was 0.50 95% CI (0.29–0.71), suggesting that the repetition of the question was moderate.

A lifestyle section containing the following five questions was excluded from the questionnaire: 1. “Due to your child’s illness, has your lifestyle changed?” – Kappa Cohen level 0.30 (0.07–0.53), agreement: 62%; 3. “Do you use stimulants?” – Cohen’s Kappa level 0.51 (0.04–0.99), agreement: 94%;4. “Do you engage in risky behavior?” – Kappa Cohen level 1, 100% agreement; 5. “Do you eat according to the principles of proper nutrition?” – Kappa Cohen level 0.50 (0.30–0.71), agreement: 66%. In the initial assumption, the lifestyle of caregivers/parents of children with CHD and/or OCD is a component that would be worth learning about in relation to the functioning of caregivers/parents of children with CHD and/or other OCD. Cronbach’s alpha for questions 20 to 24 showed poor internal consistency (0.08). The best correlations in this part of the questionnaire were obtained for questions 23 (*r* = 0.22) and 20 (*r* = 0.14). Therefore, this section on lifestyle in the assessment of the life situation of caregivers/parents of children with OCD and/or OCD was omitted.

## Discussion

The self-assessment of the life situation questionnaire for caregivers/parents of children with CHD and/or OCD developed by the authors was validated. To our knowledge, the questionnaire discussed here is the first tool dedicated to caregivers/parents that allows for a comprehensive assessment of the functioning of the caregiver of an ill child, caregivers support, emotions, and the role of spirituality in coping. The purpose of the questionnaire assessment was to find out: 1. How the disease of a child with CHD and/or OCD affects family, professional, and emotional aspects; 2. Whether spirituality (faith in God) helps in dealing with the child’s illness; and 3. In which sphere – spiritual, psychological, or material – the support of caregivers/parents of a child with CHD and/or OCD is needed. The literature lacks appropriate tools for a holistic diagnosis of the psychological, social, and emotional states of caregivers/parents of children with CHD and/or OCD, which would allow for an objective assessment of this problem. Therefore, the authors of this study attempted to develop a questionnaire assessing the life situation of caregivers/parents of children with CHD and/or OCD to better understand their needs. Understanding emotions, personal fulfillment, family situation, support, and the role of faith in God (spirituality) would be conducive to a better approach to medical care for the family of a child with CHD and/or OCD, which would translate into a direct and holistic impact on the child’s treatment and better quality health care.

The first part of the questionnaire concerned the personal lives of caregivers/parents of children with CHD and/or OCD, and included questions about family, professional, and social life. The Cronbach’s alpha (reliability coefficient of psychological tests) for this constellation of questions was 0.58, indicating close to acceptable internal consistency. After removing two questions from the analysis, this indicator increased to 0.72, which allowed us to state an acceptable level of internal consistency of the questionnaire for this range of questions. Two questions related to personal life are excluded from this section. One of them was about well-being, which was too sensitive, with only 32% (*n* = 16) of agreeing answers. The question was “How would you rate your well-being? “However, it seems useful in comparing a group of caregivers of children with cardiac diseases with that of better or worse well-being. Perhaps, the difference in responses regarding their feelings varies depending on the circumstances in which the subject finds themselves. The first answer was obtained from the child’s guardian during hospitalization when the diagnostic and therapeutic processes were underway, while the second answer (which turned out to be inconsistent with the first) was obtained when the child was at home after getting discharged from the hospital. Retaining the question about well-being, despite excluding it from the domain of personal life, would be justified in confrontation with a standardized research tool for assessing quality of life, such as the WHOQOL-BREEF (Skevington et al., 2004) or the Life Satisfaction Scale (SWLS) (Juczyński and Adamiak, 2000). The second question was excluded from the assessment of the personal lives of parents of sick children concerned with the economic and financial situation of the guardian/parent of a child with CHD and/or OCD. It is difficult to explain why the repeatability of this question turned out to be moderate, with Cohen’s Kappa value being 0.50 95% CI (0.29–0.71). There were no similar studies in the literature on the subject that would include a subjective assessment of the economic and material situations of the surveyed caregivers/parents of children with CHD and/or OCD. This might be the result of it being a “sensitive” question, especially since in this study, 36% (*n* = 18) of respondents were unemployed, of whom 70% resigned from work because the situation forced them to do so.

The second part of the questionnaire evaluates the role of faith in God (spirituality) in caregivers/parents in connection with the child’s illness. The questions in this section, after excluding the opinion about the cause of the child’s illness, showed good internal consistency with a Cronbach’s alpha of 0.83. According to the vast majority of respondents, 82% (*n* = 41) believed that their child’s illness happened by chance, 12% (*n* = 6) thought that the disease was inherited, one person believed that it was a “punishment for sins,” another person blamed their own negligence, and one person blamed the negligence of others. It is difficult to compare our research results with those of other authors because no relevant references have been found in the literature. However, there are known studies by Abu et al. (2018), which refer not to caregivers but to patients with cardiac problems, where the authors suggest that the higher the role played by faith in God (spirituality), the better the quality of life among patients with cardiovascular diseases. The questions we asked about the role of belief in God/spirituality were compared to those raised by other authors. Dalir et al. (2021) reported that in stressful situations related to caring for an ill child, prayer and recitation of the Quran reduced tension and anxiety, and strengthened caregivers and parents of children with CHD. Ahn et al. (2014) showed that both parents and children with CHD, for whom faith in God (spirituality) plays an important role, coped better with emotions than nonbelievers. The existing standardized tools concerning faith (spirituality) do not directly refer to its relationship with a child’s illness. Therefore, to provide holistic care to an ill child and its caregivers with multi-directional care (multi-faceted care), the aspect of spirituality should be taken into account in assessing the quality of functioning of caregivers/parents of a child with CHD and/or OCD. The obtained Cronbach’s alpha, which is common for both sections, was 0.66, which indicates good repeatability.

The aim of the questionnaire was to construct a tool that would be applicable not only in scientific research, but also in clinical practice. In clinical practice, the importance of training health professionals so that they possess the skills to identify and support the spiritual discomfort of patients is increasingly evident (Balboni et al., 2014). Addressing psychosocial and spiritual needs can really contribute to the improvement in the quality of life and well-being of individuals, especially of caregivers/parents of children with CHD and/or OCD. Additional descriptive parts of the questionnaire assessing the life situation of caregivers/parents of a child with CHD and/or OCD allow for extending the information to aspects related to: 1. Emotions that accompany caregivers of children with CHD and/or OCD; 2. Support received from the caregiver; 3. The caregivers’ opinions – why did the illness happened to the child; and 4. Identification of the person who mainly cares for the sick child. According to Connor et al. (2010), caregivers/parents of children with CHD experience significant stress and emotional breakdown while caring for them. According to some authors, support is crucial to ensure the best possible care for children with CHD and their caregivers/families (Mitteregger et al., 2021). Getting to know the opinions of the caregivers/parents of a child with a congenital heart defect might be useful in recognizing their attitudes towards the situation of their child’s illness. This question is compared to the studies of other authors. In the studies of Zahra Dalir et al. (2021), majority of respondents stated that their child’s illness was God’s will, and accepted it as destiny. Moreover, they hoped that their child would heal with God’s help. This indicates that the person who plays the most important role in caring for an ill child seems to be justified because, in the studies of Zahra Dalir et al. (2021), among the 40 surveyed caregivers of children with CHD, there were 27 mothers, eight fathers, two grandmothers, and three siblings of an ill child. Other authors have indicated that siblings of children with CHD are concerned about ensuring safety in the case of their brother or sister’s illness. Therefore, it is extremely important for siblings to receive psychological support (Bichard et al., 2022).

## Conclusion

The answers obtained from the questionnaire during the admission of the child and his caregivers to the hospital ward will allow for a clearer assessment of the patient and his caregivers in comprehensive medical care, which is conducive to improving the quality of healthcare. The use of a questionnaire assessing the life situation of caregivers of children with CHD and/or OCD will allow the identification of the difficulties and needs of this group to develop effective interventions to support the functioning of caregivers of children with CHD and/or OCD. The validation procedure showed that the Life Situation Assessment Questionnaire of a caregiver of a child with CHD and OCD is a reliable and uniform tool for measuring the functioning of their parents.

### Limitations

Although the results of the validation of the questionnaire for assessing the life situation of caregivers or parents of children with CHD and/or OCD confirmed that it is a tool with high reliability and repeatability, a criterion validity test should be performed and compared with the results of another study. The limitations of this study also include that the sample is made principally from women. Therefore, this study is the first step towards gaining knowledge about the life situations of caregivers and parents of children with CHD and/or OCD. This forced us to plan further research on larger groups to demonstrate the validity of the questionnaire using another standardized research tool.

## Data availability statement

The raw data supporting the conclusions of this article will be made available by the authors, without undue reservation.

## Ethics statement

The studies involving human participants were reviewed and approved by Bioethical Committee of the Medical University of Silesia in Katowice (No.: PCN/CBN/0052/KB/44/22). The patients/participants provided their written informed consent to participate in this study.

## Author contributions

EK, AM, KB, and LS: contributed to conception and design of the study. EK, AM, and LS: organized the database. KB and EK: performed the statistical analysis. EK and LS: wrote the first draft of the manuscript. EK and AM: wrote sections of the manuscript. All authors contributed to the article and approved the submitted version.

## Funding

This research was funded by the Medical University of Silesia in Katowice, Poland.

## Conflict of interest

The authors declare that the research was conducted in the absence of any commercial or financial relationships that could be construed as a potential conflict of interest.

## Publisher’s note

All claims expressed in this article are solely those of the authors and do not necessarily represent those of their affiliated organizations, or those of the publisher, the editors and the reviewers. Any product that may be evaluated in this article, or claim that may be made by its manufacturer, is not guaranteed or endorsed by the publisher.
